# Segmental sandwich osteotomy of the posterior mandible in pre-implant surgery - A systematic review

**DOI:** 10.4317/medoral.21633

**Published:** 2016-12-06

**Authors:** Georgios Kamperos, Ioannis Zografos, Fotios Tzermpos, Ioannis Iatrou

**Affiliations:** 1 DDS, MSc, MSc, Department of Oral and Maxillofacial Surgery, School of Dentistry, National and Kapodistrian University of Athens, Greece; 2 DDS, PhD, Assistant Professor. Department of Oral and Maxillofacial Surgery, School of Dentistry, National and Kapodistrian University of Athens, Greece; 3 MD, DDS, PhD, Associate Professor. Department of Oral and Maxillofacial Surgery, School of Dentistry, National and Kapodistrian University of Athens, Greece; 4 MD, DDS, PhD, Professor and Head. Department of Oral and Maxillofacial Surgery, School of Dentistry, National and Kapodistrian University of Athens, Greece

## Abstract

**Background:**

The rehabilitation of the atrophic posterior mandible with dental implants often requires bone augmentation procedures. The aim of the present study is the systematic review of the literature concerning the success rate of Segmental Sandwich Osteotomy (SSO) of the posterior mandible in pre-implant surgery.

**Material and Methods:**

Systematic review of all clinical cases and clinical studies of SSO of the posterior mandible in pre-implant surgery with a minimum follow-up of 6 months after implant loading was performed, based on specific inclusion and exclusion criteria. The search strategy involved searching the electronic databases of MEDLINE, EMBASE, COCHRANE LIBRARY, Clinical Trials (www.clinicaltrials.gov) and National Research Register (www.controlled-trials.com), supplemented by a manual search, in August 2015. In every study, the intervention characteristics and the outcome were recorded.

**Results:**

Out of the 756 initial results, only 17 articles fulfilled the predetermined inclusion and exclusion criteria. They consisted of 9 retrospective case reports or series and 8 prospective randomized clinical trials. Overall, the studies included 174 patients. In these patients, 214 SSO augmentation procedures were performed in the posterior mandible and 444 implants were placed. The follow-up period after implant loading ranged between 8 months and 5.5 years. The success rate of SSO ranged between 90% and 100%. The implant survival during the follow-up period ranged between 90.9% and 100%.

**Conclusions:**

Segmental Sandwich Osteotomy should be considered as a well documented technique for the rehabilitation of the atrophic posterior mandible, with long-term postsurgical follow-up. The success rates are very high, as well as the survival of the dental implants placed in the augmented area.

**Key words:**Segmental osteotomy, dental implant, mandible, inlay graft.

## Introduction

The rehabilitation of the atrophic posterior mandible with dental implants is often difficult due to anatomic restrictions. After the loss of teeth, the continuing bone resorption sometimes leads to an inadequate alveolar height over the inferior alveolar nerve (IAN) even for a short implant. Many bone augmentation procedures have been used including guided bone regeneration, onlay and inlay grafts, distraction osteogenesis and IAN lateralization. All of them require careful planning and great surgical skills. Furthermore, they are characterized by considerable morbidity.

The application of interpositional graft after segmental osteotomy was first introduced by Schettler, 1976, in the anterior mandible for improving the retention of a full denture (1). Clinical and histological studies confirmed the vascularization and stability of the inlay graft (2,3). Since then, many variations have been proposed and the procedure has been applied in the pre-implant surgery interventions. A lot of terms have been used to describe this bone grafting method such as “segmental osteotomy”, “sandwich osteotomy”, “sandwich technique” and “inlay technique”. In the present study, “Segmental Sandwich Osteotomy - SSO” was preferred because it incorporates the type of both the osteotomy and the graft. Yeung, 2005, was the first to treat the atrophic posterior mandible with SSO, in order to avoid the drawbacks and limitations of the other augmentation procedures (4). There are two necessary parameters: sufficient bone volume over the IAN for safe osteotomy and sufficient intermaxillary space for bone augmentation (5). Even if there are many published case reports, as well as clinical trials, for the rehabilitation of the posterior mandible with this technique, the intervention characteristics and indications are often unclear.

The aim of the present study is the systematic review of the literature concerning the success rate of SSO of the atrophic posterior mandible for placing dental implants. Moreover, the impact of the intervention characteristics on the final result is evaluated.

## Material and Methods

After an initial preliminary research, a detailed protocol was formed following the guidelines of PRISMA (Preferred Reporting Items for Systematic Reviews and Meta-Analyses) (6). The following inclusion and exclusion criteria were defined at the beginning of the systematic review:

- Inclusion criteria

1. Clinical trials, case series or case reports, using SSO of the posterior mandible in pre-implant surgery on human subjects.

2. Implant follow-up after loading should be at least 6 months, in order to assess every possible biological complication during function, rather than early failures.

3. No restriction on the publication status of the study.

4. No restriction for medically compromised patients or smokers.

5. No language restriction.

- Exclusion criteria

1. Studies not fulfilling all inclusion criteria.

2. Studies applying SSO for treatment of malpositioned osseointegrated implants.

3. Studies on animals.

4. Publications reporting the same data as later ones by the same authors.

5. Reviews

- Types of intervention

All studies applied segmental osteotomy in the posterior mandible, combined with interpositional graft and some kind of stabilization. After bone healing, dental implants were placed in the augmented site. In every study, the following intervention characteristics were recorded.

a. Number of augmented sites, 

b. Alveolar bone height over IAN,

c. Movement of the mobilized fragment,

d. Bone graft,

e. Method of stabilization, 

f. Use of a membrane or not,

g. Healing time, 

h. Number of dental implants and, 

i. Follow-up period after loading.

- Outcome measures

The cases where dental implants could be placed in the augmented site were characterized as successful. The outcome evaluation in every study involved the following clinical and/or radiographic parameters.

a. Intraoperative and postoperative complications, 

b. SSO success rate,

c. Bone gain, 

d. Bone resorption and, 

e. Implant survival at the follow-up period.

- Search strategy

The search strategy involved searching the electronic databases of MEDLINE, EMBASE, COCHRANE LIBRARY, in August 2015.

Clinical Trials (www.clinicaltrials.gov) and National Research Register (www.controlled-trials.com) were searched for unpublished studies and personal communication with the authors was attempted. The following keywords were inserted in various combinations according to the instructions of each search engine: segmental osteotomy, segmented osteotomy, sandwich osteotomy, inlay, interpositional, implant, implants, implantation, dental, alveolar, mandible, mandibular. For the MEDLINE database the following combination of words was used: (segmental osteotomy) OR (segmented osteotomy) OR (sandwich osteotomy) OR (inlay) OR (interpositional) AND (implant) OR (implants) OR (implantation) AND (dental) OR (alveolar) OR (mandible) OR (mandibular). No restriction was put on the type or language of the study. The search was supplemented by cross-checking the included articles and relevant reviews. Moreover, a manual web search was conducted for any unidentified article.

Selection criteria and data extraction

First of all, the resulting studies were checked to eliminate duplicates. The next stage involved screening the titles and abstracts on the basis of the predetermined inclusion and exclusion criteria. The final stage involved retrieving and checking the full texts of the eligible articles based on the same criteria.

Data obtained were recorded on table including:

a. Study’s characteristics (author, year of publication, type of study)

b. Patients’ characteristics (number, age, gender, smoking)

c. Details of the type of intervention (as analyzed above)

d. Details of outcome (as analyzed above)

- Quality assessment

The studies that fulfilled the inclusion criteria were qualitatively evaluated for the risk of bias (high or low risk of bias), based on the PRISMA guidelines and the simplified algorithm applied by Esposito *et al,* 2009 (6,7). Three parameters were evaluated with “YES” or “NO”:

i. Allocation concealment

ii. Outcome assessor blindness

iii. Patients’ withdrawal

For the first two parameters, “YES” corresponded to low risk of bias and “NO” corresponded to high risk of bias. On the contrary, studies with withdrawal of patients were regarded as biased, unless the authors provided reasons. Overall, the risk of bias of an included study was evaluated as low, only if all three parameters were fulfilled. It should be noted that the above parameters are designed for the quality assessment of clinical trials. In the present systematic review, the same parameters were used for the evaluation of case series / case reports, which are generally regarded as biased studies. These types of study are placed low in the pyramid of evidence-based research and there are no universally acceptable methods to evaluate their credibility.

## Results

**Figure 1:**
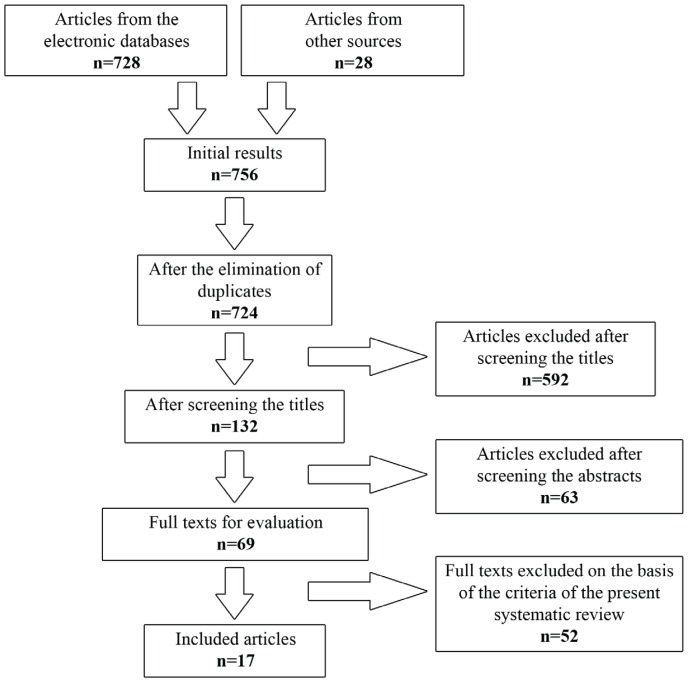
Flowchart of the systematic review.

The search yielded 756 articles; 728 from the electronic databases, supplemented by 28 from the other sources. After the elimination of duplicates and the screening of titles and abstracts, 69 full texts were retrieved for further evaluation, out of the initial results. Only 17 articles fulfilled the predetermined inclusion and exclusion criteria (8-24). The most common cause for excluding a full text was the inadequate implant follow-up period. The flowchart of the systematic review is presented in figure 1. The included articles and their characteristics are presented in  Table 1and 1 continue, and 1 continue . All included articles were published in scientific journals in English, during the period 2006-2015. They consisted of 9 retrospective case reports or series (8-24), and 8 prospective randomized clinical trials (11-15,20,21,23). Six out of the eight clinical trials compared SSO with different rehabilitation techniques, such as short implants (12-15), alveolar osteogenesis (23), and onlay bone grafts (21). The other two clinical trials compared the efficacy of various interpositional bone grafts and substitutes (11,20). Because of the significant heterogeneity of the research methods, the intervention characteristics and the outcome measures, only qualitative synthesis of the data of the included studies was performed. Meta-analysis of the data was not feasible.

**Table 1:**
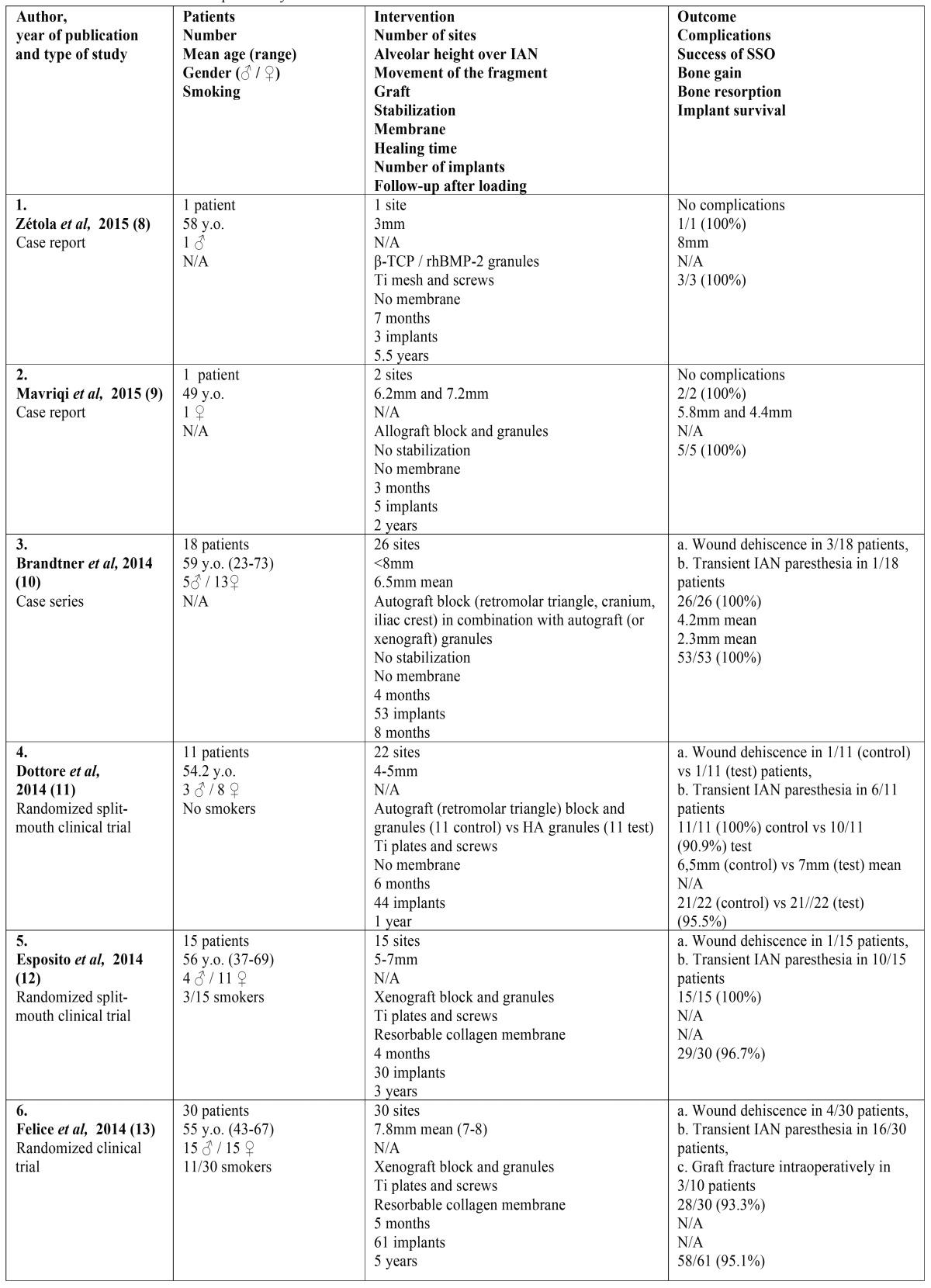
Included articles in the present systematic review and their characteristics.

**Table 1 continue:**
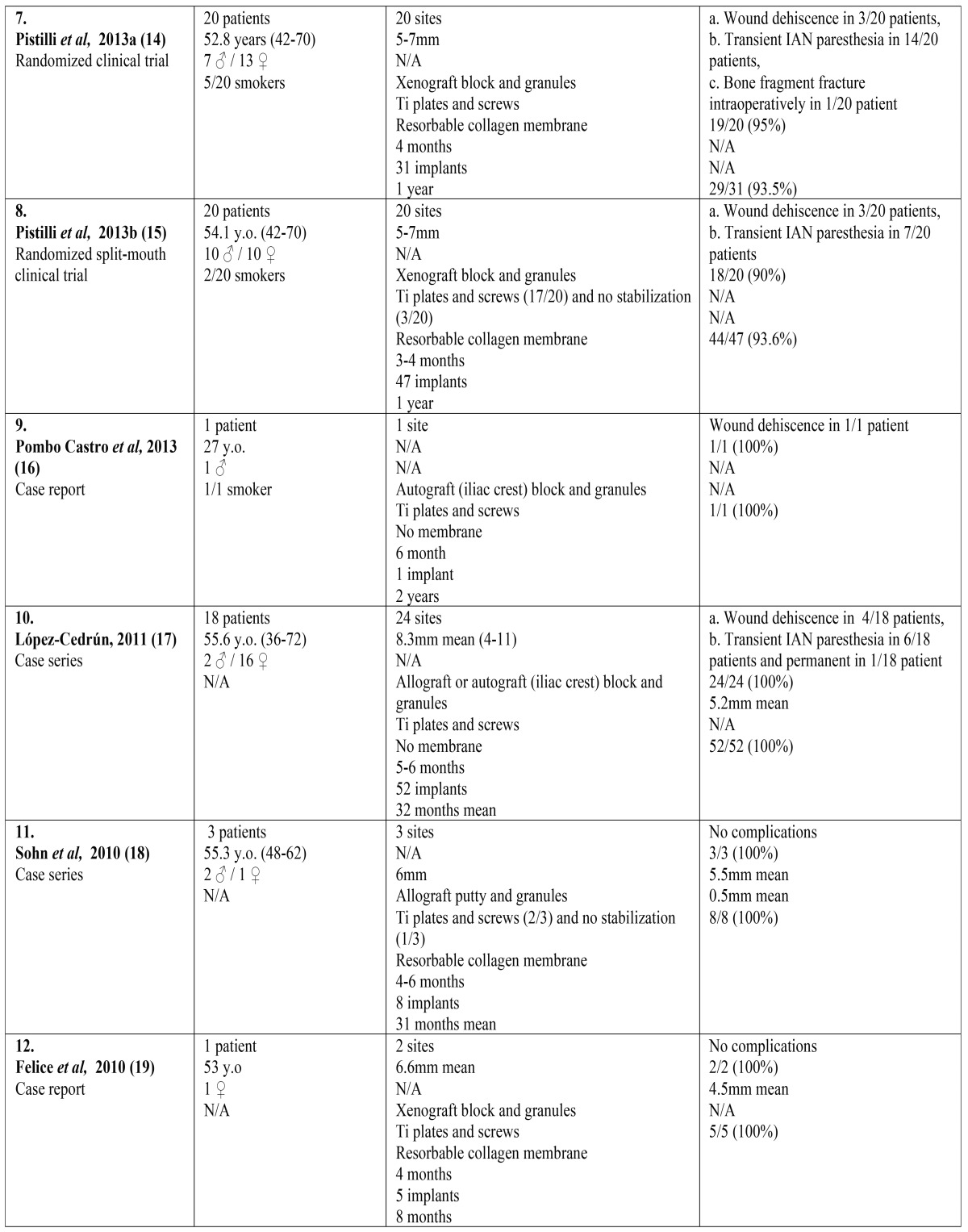
Included articles in the present systematic review and their characteristics.

**Table 1 continue:**
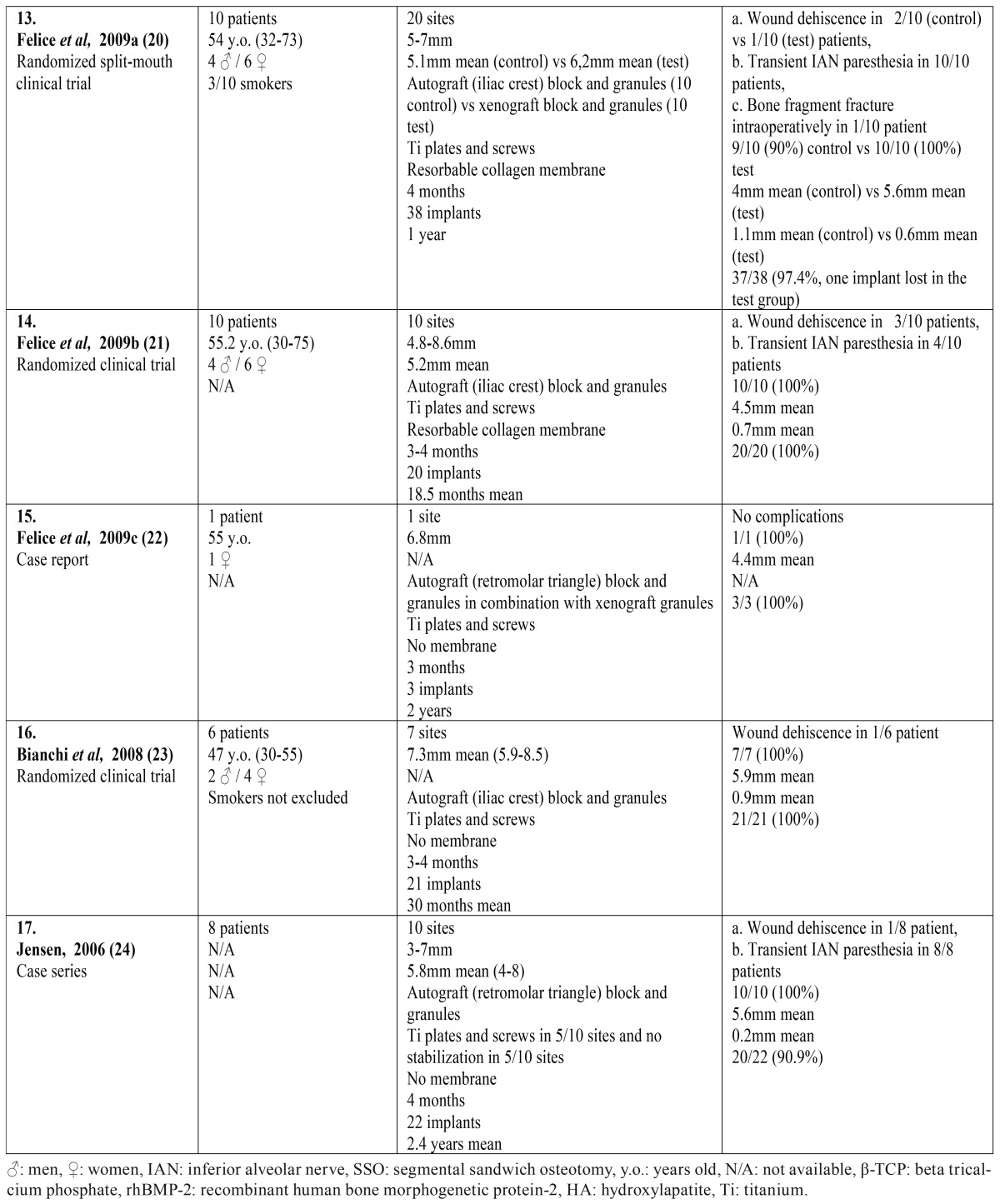
Included articles in the present systematic review and their characteristics.

- Patients’ characteristics

Overall, the 17 studied articles included 174 patients; 52 patients from the case reports or case series and 122 patients from the randomized clinical trials. There were five case reports including just one patient (8,9,16,19,22), and four case series including three to eighteen patients each (10,17,18,24). On the other side, the randomized clinical trials included six to thirty patients each (11-15,20,21,23). The age of the patients ranged between 23 and 75 years (mean age usually reported in the 6th decade), with a clear female predominance. Smokers were generally not excluded by the clinical trials, with only one exception (11).

- Types of intervention

In these patients, 214 SSO augmentation procedures were performed in the posterior mandible and 444 implants were placed. The initial height over the mandibular nerve was 4-8mm in the majority of the cases (11-15,20), and the superior movement of the bone fragment also ranged between 4mm and 8mm (10,18,20,21,24). Xenografts were most often used (12-15,19,20,22) followed by autografts (10,11,16,17,20-24), usually harvested from the iliac crest or the retromolar triangle. In the vast majority of the studies, block grafts were chosen along with granules for filling the gaps of the osteotomy (9-13,14-17,19-24). As for the stabilization of the segmented fragment, the majority of the studies chose osteosynthesis with titanium plates and screws (8,11-24), while rarely no further stabilization was used, apart from the rigidity of the bone graft (9,10,15,18,24). The augmented area was usually covered with a membrane, especially in combination with xenografts (12-15,18-21). The time of healing usually ranged between three to six months, with a slightly shorter period for the autografts in comparison with the rest graft types (8-24). The follow-up period after implant loading ranged between 8 months and 5.5 years (8-24).

- Outcome measures

The most common complication was the transient IAN paresthesia reported in 5.6% to 100% of the patients, followed by the postsurgical wound dehiscence reported in rates less or equal to 30% of the patients (10-13-15,17,20-21,23,24). Other uncommon surgical complications are the bone fragment fracture and the block graft fracture (13,14,20) Permanent IAN paresthesia is considered extremely rare (17). The success rate of SSO was very high ranging between 90% and 100%, with only five studies reporting failures (11,13-15,20), most of which were related to surgical or postsurgical complications. Wound dehiscence may be a risk factor, but does not directly lead to failure (10-13,14,15,17,20,21,23,24). On the other hand, the fracture of the segmented mandibular fragment is considered a very severe complication, certainly linked to complete failure of the augmentation procedure (14,20). Intraoperative graft fracture is also associated with increased risk of failure (14). Mean bone gain was usually 4 to 6mm (9,10,17-24), and the mean bone resorption was usually estimated between 0.2mm and 1.1mm (18,20,21,23,24). The implant survival during the follow-up period ranged between 90.9% and 100% (8-24). Last but not least, there are no indications that xenografts and bone substitutes impair the efficacy of SSO, when compared with autografts (11,20).

- Quality assessment

The nine case reports or series were evaluated as having high risk of bias (8-10,16-19,22,24). On the other hand, amongst the eight randomized clinical trials, four were assessed as having low risk (12-15), and four as having high risk of bias (11,20,21,23). The quality assessment of all included articles, based on the abovementioned parameters, is presented in  Table 2.

**Table 2:**
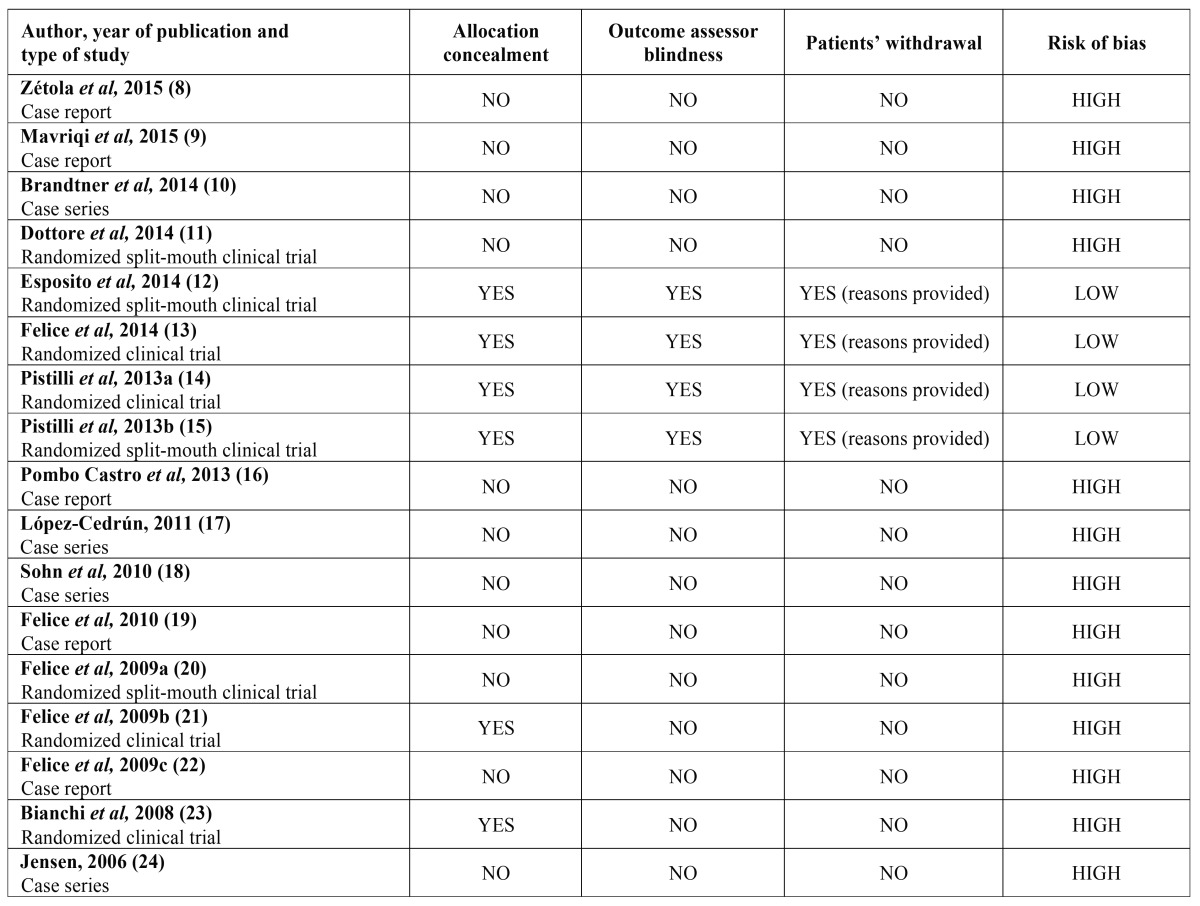
Quality assessment of the included articles.

## Discussion

Segmental Sandwich Osteotomy should be considered as a well documented technique for the rehabilitation of the atrophic posterior mandible with dental implants. According to the present systematic review, the success rate of the technique and the survival of the dental implants are very high, with long-term postsurgical follow-up. Amongst the included articles, there were many randomized clinical trials, even having low risk of bias. Furthermore, the success of SSO has been verified, not only clinically and radiographically, but also histologically by some researchers (8,19,20,22).

The intervention characteristics differ significantly among the included articles. The carefully planned osteotomy is crucial in the success of SSO. The technique is usually performed when there is 4-8mm of bone over the nerve. If the osteotomy is carried out in less than 4mm, there are two risks. Firstly, the segmented bone fragment would be thin and susceptible to fracture, especially when titanium screws are placed. It has been noted that this complication is associated with complete failure of the method (14,20). Secondly, a direct damage to IAN may occur, leading to permanent paresthesia. The use of piezosurgery is considered very useful in safely performing the osteotomy (23,25). The attachment of the soft tissues may hinder the movement of the bone fragment, but it also provides necessary vascularization for its survival (5,25). In most cases, the researchers prefer inlay block xenografts, rigid fixation and covering the augmented area with a collagen membrane. Unfortunately, there are only two randomized clinical trials comparing different bone grafts in SSO (11,20). More clinical trials are required in order to assess the impact of the intervention characteristics on the final outcome.

The outcome measures suggest that the bone gain is enough for the placement of dental implants. Moreover, the inlay grafts exhibit great stability. The loss in vertical height is attributed to a minimal resorption of the segmented fragment (21,26). As for the complications of the technique, transient paresthesia seems inevitable and is most likely caused by the traction of the buccal flap and the mental nerve (24,25). Wound dehiscence is much more uncommon. Due to the paracrestal incision, the mobile mucosa is elastic enough to close without tension (24,25). Certain researchers point that additional periosteal release incisions which may weaken the enveloping soft tissues, are unnecessary in SSO (25). Nevertheless, wound dehiscence does not always lead to failure and should be treated with topical antimicrobial agents, systemic antibiotics and sometimes local debridement (14,15,23).

The most significant parameter for choosing SSO is the initial alveolar height over the IAN. Many clinical trials have proved that short implants are preferable to complicated augmentation procedures in the restoration of the posterior mandible (12-15,27). Consequently, SSO should be reserved for cases with 4-6mm of bone over the IAN, unable to receive short implants, and with sufficient intermaxillary space. In such cases, SSO appears to be preferable to onlay block grafts and distraction osteogenesis, according to two randomized clinical trials (21,23). No trials have been published comparing SSO with IAN lateralization or guided bone regeneration. The reported advantages and disadvantages of SSO of the posterior mandible in pre-implant surgery are presented in  Table 3.

**Table 3:**
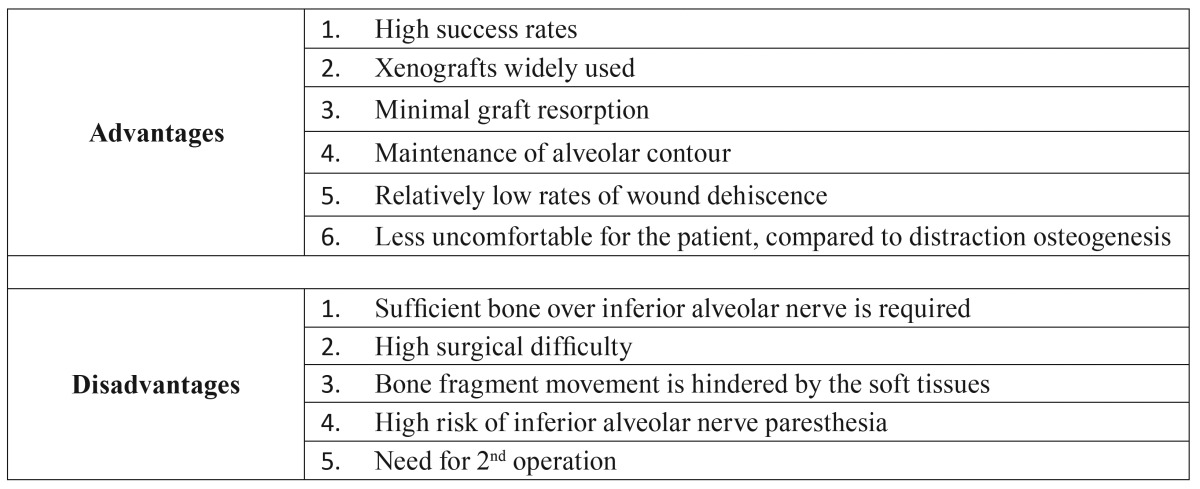
Advantages and disadvantages of Segmental Sandwich Osteotomy of the posterior mandible in pre-implant surgery.

The results of the present systematic review support the efficacy of SSO in the rehabilitation of the atrophic posterior mandible. The success rates are very high, as well as the survival of the dental implants placed in the augmented area. The impact of the intervention characteristics on the final outcome requires further research in the form of randomized clinical trials. Inlay technique has unique features and should be preferred to other augmentation procedures in selected cases.
